# New Advancements in Cisplatin-Based Treatments

**DOI:** 10.3390/ijms24065920

**Published:** 2023-03-21

**Authors:** Erica Gentilin

**Affiliations:** Bioacoustics Research Laboratory, Department of Neuroscience (DNS), University of Padua, 35122 Padua, Italy; erica.gentilin@unipd.it

Cisplatin (cis-diamminedichloroplatinum (II)) is one of the most broadly used chemotherapies worldwide. Since the unexpected discovery of its antitumor effects, huge strides have been made toward the comprehension of key determinants of the antitumoral mechanism. Despite decades of research proving that cisplatin antitumor activity is linked to DNA adduct formation and reactive oxygen species (ROS) production, the important role of inflammation is becoming increasingly apparent [1]. The use of cisplatin is indeed associated with many adverse effects, including nephrotoxicity, neurotoxicity and ototoxicity. Nephrotoxicity may be limited or prevented using several strategies [2], while neurotoxicity and ototoxicity are still lacking curative and preventive therapies [3,4].

The aim of this Special Issue is to highlight the emerging based-platinum strategies to maintain anticancer activity, avoid adverse effects and overcome cisplatin resistance.

Many different drugs and natural products reduce cisplatin toxicity [3,5]. A recent study showed that nimodipine pre-treatment of auditory cells decreases cisplatin-induced cell death in vitro [6]. Cisplatin-based chemotherapy increases the formation of reactive oxygen and nitrogen species, triggering oxidative modification of cochlear proteins and leading to the downregulation of the otoprotective transcription regulator LIM Domain Only 4 (LMO4) [7]. Nimodipine pre-treatment upregulates LMO4 and ultimately activates antiapoptotic pathways, reducing cisplatin ototoxicity [6]. In addition, emerging research has demonstrated the importance of treatment formulation beyond the treatment itself to prevent/reduce cisplatin toxicity. This is the case for dexamethasone, a hydrophobic drug which is currently used for systemic and local inner ear therapies. The use of glycerol monooleate liquid crystalline nanoparticles to deliver dexamethasone into auditory cells allows both the internalization rate and the stability of the drug within the cells to be increased [8]. In addition, the administration of these nanoparticles loaded with dexamethasone considerably diminishes cisplatin cytotoxicity in sensory cells of the inner ear [8].

Many efforts are being made to counteract cisplatin toxicities; on the other hand, extensive research has been devoted to increasing cisplatin’s effects. To achieve this goal, many formulation and delivery approaches have been developed. It has been demonstrated that nano-based cisplatin delivery systems delay drug release, extend half-life, and decrease systemic toxicity, while nanocapsules, nanogels and hydrogels improve cisplatin uptake by cancer cells [5]. In this regard, it is crucial to consider the relevance of cisplatin quantification into cancer cells, especially considering platinum resistance mechanisms. Single-cell inductively coupled plasma mass spectrometry turned out to be a precise and accurate method for quantifying cisplatin in single cells and isolated nuclei [9]. Progress in the analytical methods will lead to a better understanding of both therapeutic and undesirable cisplatin effects, resulting in the refinement of pharmacological cancer therapies. Another strategy for increasing cisplatin chemotherapeutic effects is based on combination therapy. It has been shown that diclofenac, a nonsteroidal anti-inflammatory drug, enhances cisplatin cytotoxicity in signet ring cell gastric carcinoma cells. The use of diclofenac in combination therapy with cisplatin significantly increases ROS-induced macroautophagy [10]. Aiming to improve the cancer pharmacological approach, a pilot study demonstrated, in a neuroblastoma xenograft model, the strengthening of cisplatin effects when administered as cocktail therapy with acetazolamide, a carbonic anhydrase isoform IX inhibitor, and fendiline hydrochloride, an inhibitor of several transporters involved in multidrug resistance of cancer cells [11].
